# Possible Combinatorial Utilization of Phytochemicals and Extracellular Vesicles for Wound Healing and Regeneration

**DOI:** 10.3390/ijms251910353

**Published:** 2024-09-26

**Authors:** Sachiko Koyama, Erin L. Weber, Thomas Heinbockel

**Affiliations:** 1 Department of Medicine, Indiana University School of Medicine, Indianapolis, IN 46202, USA; 2 Department of Surgery, Indiana University School of Medicine, Indianapolis, IN 46202, USA; weberel@iu.edu; 3 Department of Anatomy, College of Medicine, Howard University, Washington, DC 20059, USA

**Keywords:** phytochemicals, extracellular vesicles, regeneration, anti-inflammatory effects, possible combinatorial use, wound healing

## Abstract

Organ and tissue damage can result from injury and disease. How to facilitate regeneration from damage has been a topic for centuries, and still, we are trying to find agents to use for treatments. Two groups of biological substances are known to facilitate wound healing. Phytochemicals with bioactive properties form one group. Many phytochemicals have anti-inflammatory effects and enhance wound healing. Recent studies have described their effects at the gene and protein expression levels, highlighting the receptors and signaling pathways involved. The extremely large number of phytochemicals and the multiple types of receptors they activate suggest a broad range of applicability for their clinical use. The hydrophobic nature of many phytochemicals and the difficulty with chemical stabilization have been a problem. Recent developments in biotechnology and nanotechnology methods are enabling researchers to overcome these problems. The other group of biological substances is extracellular vesicles (EVs), which are now known to have important biological functions, including the improvement of wound healing. The proteins and nanoparticles contained in mammalian EVs as well as the specificity of the targets of microRNAs included in the EVs are becoming clear. Plant-derived EVs have been found to contain phytochemicals. The overlap in the wound-healing capabilities of both phytochemicals and EVs and the differences in their nature suggest the possibility of a combinatorial use of the two groups, which may enhance their effects.

## 1. Introduction

Damage to tissues and organs can occur for many reasons. Traumatic accidents are major incidents that clearly create significant damage. However, there are other causes of damage that are more insidious, for example, those caused by infectious diseases such as COVID-19. During the COVID-19 pandemic from 2020 to 2023, we learned that many people who contracted the SARS-CoV-2 virus unexpectedly developed conditions that lasted far beyond the acute phase of the disease, many of which are characterized by tissue/organ damage other than chronic inflammation (for example, Granholm 2023; Gonzalez-Garcia et al. 2023 [1,2]). Therapeutic agents that suppress chronic and excessive inflammation while also facilitating wound healing and regeneration may advance the treatment of tissue and organ damage caused by injury and disease.

Recently, the number of studies on extracellular vesicles (EVs) has increased significantly [3,4,5,6]. Extracellular vesicles are classified into three groups: exosome, ectosomes or microvesicles, and apoptotic bodies [7,8,9]. Exosomes are the smallest in size (30–150 nm in diameter) among these groups. They carry biomolecules such as proteins, RNA, and miRNA and are secreted from cells by exocytosis, transporting them to other locations. EVs were once thought to be debris as they were secreted/shed from cells. Now they are known to be critically involved in cell-to-cell communication. They have been found to have multiple functions, including enhancement of wound healing and regeneration and suppression of inflammation [5,9,10,11,12,13,14].

Another group of biological substances that deserve attention for their regenerative effects is phytochemicals with bioactive properties [15,16,17,18]. A large number of phytochemical compounds are known to possess anti-inflammatory effects [19] as well as enhancement of cell proliferation and cell migration, analgesic effects, anti-carcinogenesis effects, and binding affinity for specific viruses and bacteria (for example, binding affinity with SARS-CoV-2) [18,20,21]. Although EVs and phytochemicals with bioactive properties have been studied separately, only a few studies have discussed the possibility of utilizing them together, with most focusing mainly on the utilization of EVs to deliver phytochemicals.

In this review, we will focus on the biological properties of phytochemicals and EVs. We will propose their combined use with the goal of improving regeneration and recovery from damage in tissues and organs.

## 2. Wound Healing after Traumatic Injury, Surgery as a Controlled Injury, and Injury Arising from Infectious Diseases

### 2.1. Wound Healing after Traumatic Injury and Surgery

Wound healing involves three phases: inflammation, fibroproliferation, and remodeling [22]. A fourth initial phase of hemostasis is often included, in which formation of the platelet clot initiates the inflammatory process. During the inflammatory phase, macrophages, mast cells, and neutrophils accumulate at the site of injury over the course of several days. The fibroproliferative phase is characterized by an increased number of fibroblasts, collagen deposition, extracellular matrix production, angiogenesis, and epithelialization and lasts up to three to five weeks. From one month to one year after injury, the initial collagen and extracellular matrix are remodeled and efficiently reorganized into a structure and composition more similar to the native tissue. Despite the ability of tissue to repair and regenerate, the process does not always yield an optimal outcome. Over- or underactivation of any of the three phases above can lead to increased scar formation, poor wound healing, or ineffective regeneration.

Surgery is, in essence, a controlled injury. A surgical incision initiates the same wound-healing phases as injury and has the advantage of decreasing wound burden or size, reducing the distance over which cells must migrate or tissue must regenerate. Primary closure of a cutaneous wound or application of a skin graft to an expansive wound reduces the likelihood that the inflammatory phase of wound healing will become chronic and problematic. In tissues with regenerative capabilities, such as peripheral nerve, surgical repair allows for realignment of nerve architecture, bringing both sides of an injured axon in closer proximity. While this reduces the likelihood of a painful neuroma (i.e., scar), it does not eliminate time wasted through misguided axonal regeneration. For example, one week after sciatic nerve repair in mice, only 25% of axons had regenerated across the repair site, and misguided axonal sampling of multiple endoneurial tubes was more frequent than direct linear regeneration [23]. Repair of ulnar and median nerves in the mid-to-distal forearm at a distance, within which regeneration should reach muscle and return function within 12 months, resulted in highly variable results [24]. Eighty percent of median nerve repairs recovered functional, but not normal, muscle use, while 25% of ulnar nerve repairs resulted in a return of motor function. Useful sensation returned in 44% and 27% of cases, respectively. Thus, despite regenerative capabilities, good outcomes from surgical repair of some tissues or organs such as peripheral nerves will require augmentation or supplementation with, as of yet, unknown methods.

### 2.2. Damage by Infectious Diseases—An Example of Olfactory Dysfunction by COVID-19

Recent studies on the impact of COVID-19 have revealed surprisingly widespread damage to tissues and organs in some subsets of patients, such that recovery requires extensive wound healing and regeneration. For example, one of the unique symptoms of COVID-19 is the dysfunction of chemical senses. Although there are multiple possible causes for olfactory dysfunction due to COVID-19 infection, morphological damage and inflammation are considered the major etiologies. Weakening of the signaling along the olfactory pathway, i.e., from the sensory neurons in the nose to the olfactory bulb and from the olfactory bulb to the olfactory regions in the brain, due to inflammation is a well-known hypothesis for causation [18,25,26]. Although olfactory sensory neurons do not express ACE2 receptors [27,28], which SARS-CoV-2 uses as the main entry into the cells, or only sparsely express them [29], studies have shown that the olfactory epithelium becomes broadly destroyed, including the olfactory sensory neurons [30]. As the supporting cells in the olfactory epithelium express ACE2, SARS-CoV-2 and damage to the supporting cells were considered to be the inciting events, in turn causing apoptosis in the adjacent olfactory sensory neurons. Several other hypotheses have been proposed, including apoptosis of the cells due to hypoxia [31], loss of function by syncytia formation [32,33], and upregulation of genes involved in the metabolization of the odorants [34].

These studies indicate the possibility that, although there can be multiple causations for COVID-19-induced olfactory dysfunction and the major reason(s) could be different depending on the person, damage to the olfactory epithelium and chronic inflammation could be the main reason leading to olfactory dysfunction.

## 3. Phytochemicals with Bioactive Properties: Suppression of Inflammation and Enhancement of Wound Healing

### 3.1. Beta-Caryophyllene

The history of utilizing phytochemicals with bioactive properties goes back thousands of years and perhaps even before the time when records are available (for example, aloe (*Aloe vera*) [35]). However, scientific evidence of their effects at the molecular and gene expression levels started to be reported in the middle of the last century following the development of molecular biology techniques and biotechnology. We are still in great need of scientific data on phytochemicals with biological properties at the gene expression and molecular levels. For example, beta-caryophyllene (BCP) is a phytochemical present in various herbs and spices. In 2008, BCP was found to be a ligand of cannabinoid receptor 2 (CB2), but not a ligand for cannabinoid receptor 1 (CB1) [36]. Various chemical compounds activate both CB1 and CB2, and, since activation of CB1 has psychoactive effects, the finding that BCP activates only CB2 was exciting in terms of potential drug development.

CB2 was first identified in the 1990s following the identification of the CB1 receptor [37]. CB2 is primarily expressed by leukocytes [38] and was thus considered as a therapeutic target for immunomodulation and treatment of neuropathic pain [39]. Studies have shown that BCP stimulates the release of endogenous opioid β-endorphin, exerts an analgesic effect in mouse models of inflammatory and neuropathic pain [40], and reduces anxiety in mice [41]. BCP was also noted to possess antioxidant activity in rats [42] while reducing the expression of stress-related genes in *Caenorhabditis elegans* [43]. BCP suppressed inflammation [36] and at least two types of regulated cell death: necroptosis in mice [44] and apoptosis in rats [45]. These effects suggest that, in terms of wound healing, BCP could facilitate the switch from the inflammatory stage of wound healing, which is the first stage in the process of wound healing, to the cell proliferation stage, and thus enhance wound closure. In our previous study of murine cutaneous wounds, application of BCP directly to the wound surface enhanced cutaneous re-epithelialization [17]. When these skin samples were harvested 17 to 18 h after exposure to BCP, during the inflammatory stage of the wound-healing process, they showed significantly up-regulated expression of keratins and keratin-associated protein (*Krtap*) and down-regulation of expression of pro-inflammatory cytokine genes IL1β and IL6. Hair follicle bulge markers *Gli1*, *Lgr5*, *Sox9*, and hair follicle infundibulum marker *Lrig1* were also upregulated, suggesting that exposure to BCP can stimulate hair follicle stem cells, a source of epithelialization in expansive wounds [17]. Although healing of adult mammalian wounds typically culminates in tissue repair with scarring rather than organ regeneration with perfect restoration of structure and function, murine cutaneous wounds treated with BCP displayed a possibility of full regeneration [17]. The TREM1 signaling pathway, an inflammation pathway, was suppressed and the signaling pathways related to cell proliferation and migration such as the sonic hedgehog pathway, planar cell polarity pathway, fibroblast growth factor signaling pathway, and Wnt/β-catenin pathways were significantly activated compared to the control group [17]. In a later stage, i.e., 4 days after wounding, the number of apoptotic cells in the wound bed was found to be significantly lower when exposed to BCP [17]. These results suggested the highly promising possibility that BCP facilitates recovery from injuries and morphological damage in tissue and organs.

In our previous study [17], we showed that BCP enhances wound healing in mice through multiple pathways. Several other studies have reported such multi-pathway activation and the involvement of BCP in the effects. One such pathway is through the activation of a nuclear receptor family, the peroxisome proliferator-activated receptor (PPAR) pathway. There are three sub-types of PPAR, i.e., α, β, and γ, and BCP is known to activate PPAR-α directly in vitro [46] and PPAR-γ indirectly through activation of CB2 in mice and rats [47,48,49]. Multiple pathways, or mechanisms of action, are considered to produce the anti-inflammatory effects of PPAR receptors. For example, they are known to suppress the nuclear factor kappa-light-chain-enhancer of activated B cells (Nf-kB) [50], which is a transcription factor involved in immune responses to stress factors and induces the expression of pro-inflammatory cytokines and chemokines [51,52]. PPAR blocks the mitogen-activated protein kinase (MAPK) signaling cascade [53], which is associated with pro-inflammatory responses to environmental stress factors [54]. As such, BCP suppresses two major pathways involved in inflammation and enhances cell proliferation and cell migration, making it a strong candidate for facilitating regeneration.

### 3.2. Other Phytochemicals

BCP is not the only phytochemical compound with bioactive properties, and CB2 is not the only signaling pathway or mechanism that can be involved in anti-inflammatory effects, as we have discussed briefly above. Phytochemicals offer several benefits as medical treatments. To avoid toxicity at high doses, multiple phytochemicals may be used in combination, potentiating their anti-inflammatory or wound-healing effects. As we saw with BCP, multiple signaling pathways may be activated, producing potentially synergistic effects.

The benefit of using phytochemicals as supplements for medical treatments to facilitate recovery is their multi-function. Because of the large number of chemical compounds with anti-inflammatory effects, the possibility exists of combinatorial use of multiple phytochemicals. Another reason is, as we saw in the example of BCP, chemical compounds can activate multiple signaling pathways and possibly produce synergistic effects. Another benefit of utilizing phytochemicals is that we can choose the most appropriate one depending on the goals. Some phytochemicals have been studied for their highly specific effects. For example, carvacrol and geraniol [55,56] display binding affinity for the SARS-CoV-2 receptor-binding domain (RBD) of the spike glycoprotein (S-protein) and may suppress infection and lessen COVID-19 symptoms. The green tea polyphenol Epigallocatechin Gallate (EGCG) and epicatechin bind and inhibit proteases involved in viral replication such as 3-chymotrypsin-like cysteine protease, 3CLpro (also known as main protease, Mpro), and papain-like protease (PLpro) (EGCG and epicatechin for 3CLpro [57,58] and epicatechin for PLpro [58]). For other diseases, whether infectious or not, or for recovery from injuries and/or surgeries, it may be possible to select the phytochemicals suitable for the disease conditions involved and the effects desired. In addition, the personal level of a disease condition requires consideration, i.e., precision medicine (a concept of “tailoring disease prevention and treatment that takes into account differences in people’s genes, environments, and lifestyles” (from https://www.fda.gov/medical-devices/in-vitro-diagnostics/precision-medicine accessed on 18 September 2024). Started by President Obama’s State of the Union Address in 2015, the goal of precision medicine is to “pioneer a new model of patient-powered research that promises to accelerate biomedical discoveries and provide clinicians with new tools, knowledge, and therapies to select which treatments will work best for which patients” (https://obamawhitehouse.archives.gov/the-press-office/2015/01/30/fact-sheet-president-obama-s-precision-medicine-initiative accessed on 18 September 2024)), depending on existing conditions, genetic conditions, sex and age differences, psychological factors, and other conditions.

Other phytochemical effects are indirect in nature, in that they are not directly caused by activation or inhibition of certain receptors. Carnosic acid is a diterpene chemical compound included in, among others, rosemary (*Rosmarinus officinalis* L.) and salvia (*Salvia officinalis* L.). Carnosic acid is not a CB2 ligand. In vitro and in vivo assays and proteomic approaches utilizing bioinformatics have demonstrated anti-inflammatory effects [59,60,61,62], and the anti-inflammatory effects are mediated through both direct and indirect action. The direct pathway is thought to suppress the production of pro-inflammatory cytokines and chemokines [62], whereas the indirect effects were found to be mediated through increased transcription of antioxidative enzymes in in vitro and in vivo assays [63]. An increase in reactive oxidative species (ROS) causes a decrease in their elimination, which leads to chronic inflammation (oxidative stress). Antioxidants suppress inflammation through multiple mechanisms, for example, by scavenging of reactive oxygen species (ROS) and the inhibition of enzymes related to oxidation [64]. Thus, an increase in antioxidative enzyme quantity or activity suppresses inflammation. Some studies argue that these indirect effects on inflammation are stronger than direct activation of receptors because the signaling pathway is more stable and amplified by the production of multiple types of antioxidative enzymes [63], including ‘phase 2 enzymes’ heme oxygenase-1 (HO-1), NADPH quinone oxidoreductase 1 (NQO1), and γ-glutamyl cysteine ligase (γ-GCL) [63].

Curcumin is another well-studied phytochemical compound. Curcumin is present in turmeric (*Curcuma longa*) and has been long known to improve wound healing, in addition to various other effects, including anti-inflammatory (reviews [18,65]), anti-carcinogenic (in vitro models [66,67]), and anti-oxidant effects, as well as suppression of neuropathic pain (mice model [68]). These effects are very similar to the effects of BCP, but curcumin does not activate the CB2 receptor. Potential receptors of curcumin (Table 1) include PPARγ (a review [69]), aryl hydrocarbon (AhR) (in vitro assay [70]), and TRPA1 (in vitro assay [71]). Outlined in a review by Zhou et al. (2011) [72], multiple pathways and mechanisms of action are believed to be involved in the anti-inflammatory effects of curcumin. 

Table 1 summarizes some phytochemicals with known anti-inflammatory effects. As we have presented a more complete list of phytochemicals elsewhere [18], here, we focus on the signaling pathways which mediate the anti-inflammatory effects. 

**Figure 1:**
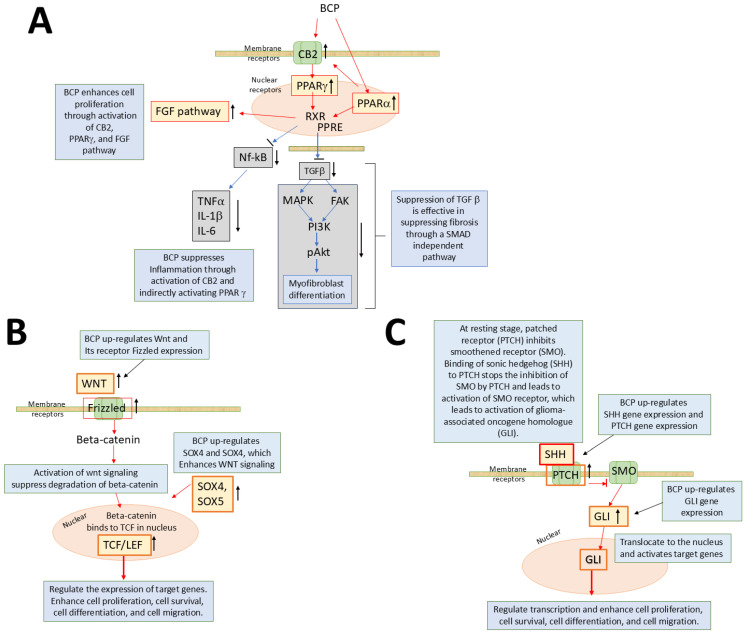
Models for BCP-induced wound healing and regeneration. (**A**) BCP is a ligand of CB2 [36] and PPARα [46]. Activation of PPARα increases the expression of CB2 [158] as well, which may further enhance the effects of BCP. Crosstalk between CB2 and PPARγ induces activation of PPARγ [47,48,159], and the activation of both PPARα and PPARγ leads to their interaction with RXR and formation of a heterodimer complex, which will bind with PPRE. Activation of PPARα and PPARγ has anti-inflammatory effects and neuroprotective effects by suppressing NFkB, IL-1β, IL-6 [158,160,161]. It also suppresses myofibroblast differentiation and, thus, is expected to decrease fibrosis during wound healing [162]. (**B**) BCP also up-regulates WNT (especially WNT5a, WNT11, and WNT10b) [17], Frizzled receptor, SOX4 and SOX5, and TCF/LEF, activating WNT signaling [17] and increasing cell proliferation and cell migration. (**C**) BCP activates SHH signaling [17] by up-regulating SHH and GLI and enhances cell proliferation and migration, enhancing regeneration. AKT: protein kinase B, FAK: focal adhesion kinase, FGF: fibroblast growth factor, IL-1β: interleukin 1β, IL-6: interleukin 6, MAPK: mitogen-activated protein kinases, NFkB: nuclear factor kappa B, PI3K: phosphatidylinositol-3-kinase, PPRE: PPAR response element of target genes, RXR: retinoid X receptor, TGFβ: transforming growth factor β, TNFα: tumor necrosis factor-alpha, SOX: SRY-related high-mobility-group-box, TCF/LEF: T cell factor/lymphoid enhancer-binding factor, SHH: sonic hedgehog, PTCH: patched, SMO: smoothened receptor, GLI: glioma-associated oncogene homolog. Red arrows in the signaling pathways indicate up-regulation or activation, and blue arrows in them show down-regulation or suppression.

Table 2 summarizes the receptors and channels affected by phytochemicals. Many phytochemicals activate or suppress multiple targets, including multiple targets within an individual signaling pathway as well as distinctly separate signaling pathways. This multiple target capacity likely explains the wide biological actions of phytochemicals. Figure 1 depicts possible mechanisms of action of enhanced tissue regeneration or wound healing of the phytochemical compound BCP’s activation of multiple receptors.

**Table 2:** Some examples of receptors and channels activated or inhibited by phytochemicals.

Action	Targets: Receptors/Channels/Other Targets	Phytochemical Compound	Triggers/Function/Reference
Activation	Cannabinoid receptor 2 (CB2)	cannabigerol, β-caryophyllene, citral (those that activate CB1 as well are excluded)	Activation of CB2 has anti-inflammatory, analgesic, and anxiolytic effects (in vivo, in vitro, and reviews [36,122,123,124])
	Transient receptor potential channel ankyrin 1 (TRPA1)	Allicin, allyl isothiocyanate, bradykinin, cannabigerol, carvacrol, cinnamaldehyde, citral, curcumin, diallyldisulfide, eugenol, ligustilide, D-limonene, linalool, linalyl acetate, osthole, methylsalicylate, paclitaxel, THC, and others	Thermosensation, mechanosensation, chemosensation, pain, inflammation [125]; activated by multiple different mechanisms [126]; activation by phytochemicals induces changes in hormone secretion, neuropeptide/neurotransmitter release [127] (reviews)
	Transient receptor potential channel melastatin 8 (TRPM8)	L-Carvone, 1,8-cineole (eucalyptol), citral (geranial), eugenol, geraniol, hydroxyl-citronellal, icilin, isopulegol, linalool, 1-menthol, menthyl lactate, and others	Activated by cold [126], voltage, pressure, cooling compounds (menthol, icilin), hyperosmolarity [127,128] (reviews)
	Transient receptor potential channel vanilloid 1 (TRPV1)	Allicin, β-caryophyllene, camphor, cannabidiol, cannabigerol, capsaicin, carvacrol, citral, eugenol, evodiamine, gingerol, 1-menthol, piperine, resiniferatoxin, thymol, vanillin, and others	Activated by heat, suppresses pain [127] (review)
	Transient receptor potential channel vanilloid 3 (TRPV3)	Borneol, camphor, carvacrol, carveol, eugenol, thymol, and others	Warnth sensor, skin sensitization [127]; analgesic (review)
	Peroxisome proliferator-activated receptor (PPAR)	Apigenin, berberine, β-caryophyllene, cannabigerol, carnosic acid, carvacrol, catechins, curcumin, eugenol, hesperetin, isorhamnetin, kaempferol, naringenin, phloroglucinol, phlorotannins, procyanidin, quercetin, resveratrol, rutin, shogaol, terpinen-4-ol, and others	Regulation of energy homeostasis, insulin sensitization, glucose metabolism, and fatty acid metabolism [129,130]. Antioxidant, anti-inflammatory, anti-obesity, and other effects (reviews)
	GABA/5-HT	Apigenin, carvacrol, citral, geraniol, kaempferol, d-limonene, linalool, linalyl acetate, luteolin, methyl eugenol, obovatol, oleanolic acid, querecetin, rosmarinic acid, rutin, santin, saponins, tannins, terpinen-4-ol	Acts as inhibitory neurotransmitter and reduces excitability. Phytochemicals in jujuba seeds regulate GABA and 5-HT receptors to exert their anxiolytic effects [131]. A review [132]
	ER*α*	Apigenin, coumestrol, genistein, liquiritigenin, resveratrol	Phytochemicals with binding affinity with ERα receptor (phytoestrogen) produce estrogenic effects [133]. A review [134]
	Adenosine A2A	D-limonene [102]	Adenosine A2A regulates, for example, immune responses cardiovascular function, sleep regulation, and others [135]. A review [102]
	67 kDa laminin receptor (67LR)	EGCG [91,136,137]	Involved in cell adhesion, migration, proliferation, and survival. A review [136]
	Aryl hydrocarbon receptor (AhR)	Curcumin potential ligand [70]	Detoxication of xenobiotic compounds [138]. In vitro sudy using rat astrocytes [70]
	GPR55	Curcumin [87]	Increases intracellular calcium. In vitro study [87]
Suppression	Kv (potassium channels) (removed the specific type)	Citronellol, citral, EGCG, geraniol, linalool, loureirin B, luteolin, naringenin, quercetin, resveratrol	Involved in cell proliferation, hormone secretion, neurotransmitter release, and others (a review and in vitro study [139,140])
	Cav (calcium channels) (removed the specific type)	α-besabolol, betulinic acid, camphene, cannabidiol, curcumin, eugenol, gingerol, shogaol, linalool, quercetin	Review [141]
	Nav (sodium channels) (removed the specific type)	Gingerol, imperatorin, lappaconitine, methyl eugenol, narirutin, peimine, shogaol	Activation of neuronal signaling related to the perception of pain (reviews and an in vivo study [141,142,143])
	Nuclear factor kappa-light-chain-enhancer of activated B (NF-kB)	Apigenin, caffeic acid, curcumin [88], EGCG, gallic acid, genistein, 6-gingerol, quercetin, resveratrol	Reviews [144,145]
	Toll-like receptors (TLR)	Curcumin, EGCG, helenalin, cinnamaldehyde, sulforaphane, but not resveratrol	Detect pathogens and activate proinflammatory pathways to eliminate pathogens; overactivation is involved in inflammatory diseases (reviews and an in vitro study [144,146,147,148,149,150])
	Tank binding kinaze 1 (TBK1)	Resveratrol, EGCG, luteolin, quercetin, chrysin, eriodictyol [146]	Inducer of type 1 interferons and involved in innate immunity signaling (a review [151])
	Nucleotide-binding oligomerization domain proteins (NOD) 2	Curcumin, parthenolide, helanalin, but not resveratrol and EGCG	Detect pathogens and activate proinflammatory pathways (a review [146])
	Tumor necrosis factor α (TNFα)	Capsaicin, curcumin, EGCG, kaempferol, naringenin, piperine, quercetin resveratrol, retinoic acid, rosmarinic acid, rutin, 6-shogaol, theaflavin [152]	Reviews [152,153]
	Rat sarcoma (Ras)	Oridonin, perillyl alcohol	GTP-binding protein which stimulates various vital cellular processes such as cell proliferation and survival, differentiation, and others Activation triggers a pathway of RAS -> RAF -> MEK -> ERK-1/2 (review [154])
	Mitogen-activated protein kinase (MAPK)	Caffeic acid, EGCG, kaempferol, magnolol, perillyl alcohol, quercetin, resveratrol, ursolic acid	Review [155]
	Adenosine A2A	Caffeine [156], flavonoids such as galangin [157]	

### 3.3. Combinatorial Use of Phytochemicals

Despite the well-known beneficial effects, there are several issues that require consideration and improvement in order to utilize phytochemicals: (1) many are hydrophobic, which reduces bioavailability and requires larger concentrations to deliver a sufficient amount to produce an effect; (2) some of them are chemically unstable and the oxidated products may have a different or less bioactive nature, and/or may cause increased allergic reactions; and (3) some become toxic at very low concentrations and require caution for therapeutic use.

To overcome these problems, phytochemicals which share the same targets (for example, sharing the same biological actions on the same receptor/channel) may be used in lower concentrations together. In this way, it is possible to enhance the activation of the receptors/channels without increasing the concentration of one chemical compound. The bioactive effects may also be enhanced by the activation of the receptors/channels that they do not share. One such example is the combined use of curcumin with other phytochemicals [163,164,165]. Although the biologically active properties of curcumin, including beneficial effects on health, are well known, the hydrophobic nature of curcumin hinders the efficacy. Combinations of, for example, curcumin with piperine, resveratrol, EGCG, and quercetin are reported to show synergistically higher anti-cancer effects [164] (Table 3). The combinatorial use of EGCG and curcumin has been reported to modify the drug-resistant nature of cancer cells, increasing the intracellular levels of cancer chemotherapy agent doxorubicin in MCF-7 cancer cells [166]. 

Another combinatorial use of phytochemicals is their use in combination with extracellular vesicles (EV) to enhance delivery, which we will discuss in the next section.

## 4. Extracellular Vesicles (EVs) for Wound Healing

### 4.1. Biology of EVs

Studies on EVs have increased exponentially during the last couple of decades. EVs have been shown to enhance wound healing in corneal endothelial cells [178], corneal epithelia [179], and skin [180,181], as well as to enhance angiogenesis in gliomas [182] and ischemic disorders [183]. The effects include suppressing inflammation, promoting fibroblast proliferation and migration, supporting re-epithelialization, promoting collagen synthesis and angiogenesis, and suppressing scar formation by suppressing myofibroblast differentiation. EVs are becoming a promising agent in tissue and wound healing.

EVs can be isolated from various sources, including mammalian cells, tissues/organs, body fluids such as serum and saliva, and plants. Of the cell lines, mesenchymal stem cells (MSC) have been often reported to have positive effects on nerve regeneration (for example, see a review on peripheral nerve regeneration, Dong et al. 2019 [184] and Lim et al. 2023 [185] (See Table 4). Many studies use EVs from adipose-derived mesenchymal stem cells, which are now called adipose-derived stem cells without specifically mentioning mesenchymal stem cells [186]. EVs isolated from adipose-derived mesenchymal stem cells (ADMSC-EV) loaded on hydrogel improved the regeneration of peripheral nerves [187] and attenuated inflammation after tendon injuries [188]. Studies on the effects of EVs isolated from bone marrow-derived mesenchymal stem cells (BM-MSC) improved corneal wound healing [10] and foot ulcer wound healing [189]. Studies have shown that EVs from Schwann cells (SC-EV) promote peripheral nerve regeneration [190]. Studies using EVs isolated from muscles (MUS-EV) have demonstrated that exposure to MUS-EVs enhances motor neuron regeneration [191].

There are various biological factors known to affect the protein and gene expression profiles of EVs. The EVs isolated from MSC cells have a stronger impact on regeneration than other cells, and they are known to produce a larger amount of EVs [192,193]. The quantity of EV released and the protein/gene content of EVs isolated from the same cells may change depending on conditions of stress or injury. Studies on kidney diseases and kidney injuries have shown changes in the markers contained within or on EVs, such that they could be used in the diagnosis of kidney conditions [194]. EVs isolated from denervated skeletal muscle following peripheral nerve injury have a stronger impact on motor neuron regeneration than do EVs isolated from uninjured, innervated muscle [191].

In the previous section, we described the effects of phytochemicals, chemical compounds of plant origin. Do EVs from plants also have therapeutic potential and is there any relationship between EVs and phytochemicals? The discovery of the plant-derived EV/nano-particle (PDEV) came about 20 years later than that of mammalian EVs [5,6,195]. Few studies have focused on the effects of PDEV in wound healing or regeneration. Many of the studies on the effects of PDEV have demonstrated anti-cancer and anti-inflammatory effects (Urzì et al. 2021 [196], Karamanidou and Tsouknidas 2021 [197], Barathan et al. 2024 [198] for review). The types of plants used in the studies on the anti-cancer effects of PDEV are, for example, tea leaf [199], cannabis [200], lemon [201], and ginseng, which all showed positive anti-tumor effects [202]. Studies using ginger-derived [203] and grapefruit-derived EVs [204] showed that PDEV can suppress colitis [204] and colitis-related cancer [203]. Compared to mice with experimentally induced colitis created by placement of 2% dextran sulfate sodium (DSS) in their drinking water, mice that received grapefruit-derived PDEV by gavage for 7 days prior to receiving DSS in water showed significantly lower expression of TNFα and IL-1β in intestinal macrophages and the colon [204]. In a study by Zhang et al. (2016) [203], ginger-derived PDEV was administered orally when 1.5% DSS was added to the drinking water. Mice that received ginger-derived PDEV showed significantly lower expression of intestinal inflammation biomarker lipocalin-2, which was as low as the control group without DSS treatment, significantly lower expression of pro-inflammatory cytokines TNFα and IL-1β, and higher expression of anti-inflammatory cytokine IL-10 [203]. A recent review showed a summary of the effects of EV from ginger, turmeric, and tea leaf on suppressing pro-inflammatory biomarkers in colon tissues, suggesting possibilities of utilization in inflammatory bowel disease [205].

EVs isolated from ginger contain phytochemicals such as 6-gingerol and 6-shogaol [203], which are well known for their anti-inflammatory, anti-cancer, and anti-oxidation effects, suppressing the TLR4 signaling pathway [147]. Similarly, EVs from grapefruit contain naringenin, which is also a well-known phytochemical compound with anti-inflammatory and analgesic effects [204,206,207]. This suggests that the effects of PDEV could be attributed, at least in part, to the phytochemicals included in them. Considering the large number of studies showing the effects of phytochemicals on wound healing and regeneration, there is a possibility that PDEV shows similar positive effects. Table 4 shows some studies using EVs from mammals and plants, describing their effects on regeneration and the effects of PDEV on cancer, wound healing, and inflammation.

**Table 4:** Examples of the effects of EVs on regeneration and examples of the studies on the effects of PDEV.

Source		Effects Found	References
Mammal/Plant	Cell/Tissue/Organ		
Mammal	Cell (MSC)	Promoted corneal epithelial cell proliferation, migration in vitro, and wound healing in vivo; umbilical cord MSC	[179]
	Cell fragments (Platelet)	Corneal endothelial cells showed increased viability and enhanced wound healing, adhering rate, and proliferation markers when exposed to EV from platelets	[178]
	Cell (MSC)	EV enhanced in vivo cutaneous wound healing and suppressed apoptosis; adipose-derived MSC	[180]
	Cell (MSC)	EV enhanced cell proliferation and migration of human corneal epithelial cells, p44/42 MAPK pathway was activated; they suppressed inflammation and suppressed upregulation of α-SMA (fibrosis)	[10]
	Cell (MSC)	Review; accelerated wound-healing process; suppressed inflammation; promoted vascularization, cell proliferation, and migration	[208]
	Cell (MSC)	Review; improved axon extension, apoptosis inhibition, Schwann cell proliferation, and neuroregeneration in nerve regeneration	[185]
	Cell (MSC)	Review; improved axon extension, promoted axon regeneration by delivering neurotrophic factors, suppressed neuroinflammation, mediated vascular regeneration	[184]
	Cell (ASC)	ASC-derived EV loaded in thermosensitive hydrogel enhanced Schwann cell migration and proliferation, as well as axon extension in in vitro and nerve repair in vivo (rat)	[187]
	Cell (ASC)	ASC-derived EV loaded on collagen sheet suppressed early inflammatory response and post-repair tendon gap and facilitated collagen formation	[188]
	Cell (Sch)	EV from Schwann cells that received mechanical stimulation by exposure to magnetic field had stronger influences of enhancing axonal growth in vitro and nerve regeneration in vivo than EV from Schwann cells without mechanical stimulation	[190]
	Cell (Muscle)	EV from muscle cell line enhanced neurite growth and survival	[209]
	Tissue (Muscle)	EV from denervated muscle improved the motor neuron’s ability to correctly project back to the original terminal muscle branch, showing enhanced regeneration accuracy	[191]
Plant	Tea leaf	Suppressed breast tumor cell cycle by increasing ROS, triggering mitochondria damage and causing apoptosis in tumor cells	[199]
	Review	Review on the effects of plant-derived vesicles on cancer and inflammation	[196]
	Lemon	EV from lemon juice suppressed tumor cell proliferation	[201]
	Ginseng	EV from ginseng increased apoptosis in mouse melanoma cells	[202]
	Cannabis	EV from cannabis suppressed cell proliferation and increased apoptosis, suppressing tumor growth	[200]
	Ginger	EV from ginger suppressed acute colitis, facilitated intestinal recovery, and suppressed colitis-related cancer. EV contained a large amount of 6-gingerol and 6-shogaol	[203]
	Grapefruit	EV from grapefruit suppressed dextran-sulfate sodium (DSS)-induced colitis in mice	[204]

MSC: mesenchymal stem cells; ASC: adipose-derived stem cells; Sch: Schwann cells.

### 4.2. The Role of EVs as Drug Carrier

The fact that PDEVs contain phytochemicals with bioactive properties indicates that they can function as drug carriers for delivery. If so, what about the mammalian EVs? Do they also carry molecules with anti-inflammatory and pro-regenerative effects?

A study using exosomes, one of many types of EVs, from adipose-derived stem cells (ADSC) found that a long noncoding RNA called metastasis-associated lung adenocarcinoma transcript 1 (MALAT1) contained in the exosomes enhanced cell proliferation and cell migration and suppressed apoptosis in cutaneous wounds, whereas exosomes without MALAT1 lacked those effects in an in vitro model of wound healing [210]. The study also showed that MALAT1 triggers the Wnt/β-catenin pathway, enhancing the expression of miR-124 [210]. The effects of exosomes diminished when miR-124 was suppressed by anti-miR-124 [210]. The role of MALAT1 in regeneration was also reported in an in vivo study on traumatic brain injury in rats [211]. Exosomes isolated from human ADSC containing or lacking MALAT1 were injected into rats with brain injury. The rats treated with exosomes with MALAT1 showed significantly lower expression of inflammation markers IL-1β and TNFα and significantly better recovery of motor behaviors [211]. A recent study of MALAT1 showed that the effects are not necessarily mediated by miR-124. A study using exosomes from human keratinocytes showed that MALAT1 enhanced wound healing, mediated by binding to miR-1914-3p and upregulating MFGE8 [212]. According to Ross (2021) [213], “a given miRNA can regulate numerous mRNAs, and consequently miRNAs function as master regulators of the molecular status of the cell”. The review paper focuses on cutaneous wound healing and lists eight different miRNAs that regulate migration, proliferation, differentiation, inflammation, and wound closure [213]. Other studies focused on muscles. Mizbani et al. (2016) [214] have shown that miR-501 is involved in muscle regeneration, and inhibition of miR-501 suppresses the expression of myosin isoforms during muscle regeneration [214]. Another study by the same group showed that there are several miRNAs that are involved in inhibitory roles [215]. Luca et al. (2020) have shown that a group of five miRNAs together inversely correlated with the activation of focal adhesion kinase, AKT, and p38 mitogen-activated protein kinase (MAPK), and improved myotube formation [215]. In their in vivo study using mice, they found that inhibition of these miRNA networks enhanced the mass of regenerated muscle after injury. The miRNAs included in the five miRNAs were miR-29a, let-7, miR-125b, miR-199a, and miR-221 [215]. These studies suggest that there are miRNAs with inhibitory or enhancing roles in regeneration. How do exosomes affect axon regeneration of peripheral nerves? A recent study has shown that exosomes isolated from primary fibroblasts (FB-EV) from injured sciatic nerves with or without exposure to chitosan oligosaccharides enhanced axon extension and regeneration of injured sciatic nerves [216]. In this study, they found that transcription factor AP-2 γ (TRAP2C), which is a protein included in FB-EV isolated from sciatic nerves exposed to chitosan oligosaccharides, has a key role in facilitating regeneration, and it was mediated through the miR-132-5p/CAMKK1 signaling pathway [216]. In another study, exosomes isolated from ADSC contained miR-22-3p, which promoted proliferation and migration of Schwann cells and enhanced axon extension of dorsal root ganglion neurons in vitro [217]. Many studies have shown that exosomes isolated from MSC (for example, Liu et al. 2019 [218] and Sun et al. 2018 [219]) have anti-inflammatory effects in the nervous system as well. These studies indicate that (1) various types of microRNAs are involved in regeneration, (2) some function to suppress regeneration and some function to enhance regeneration, (3) multiple signaling pathways are involved, (4) they function to suppress inflammation, and (5) microRNAs appear to be specific for certain types of cells or tissue/organs. Even in the same region of injury, for example, a hand, different types of tissues are damaged, such as skin, muscle, peripheral nerves, and various cell types, which means that there are multiple targets for whole and full regeneration. It could be that EVs function as cargo ships that carry different types of microRNAs and deliver them to their target locations where they can carry out their specific roles.

### 4.3. The Possibility of Utilizing EVs in Drug Delivery

As described above, EVs can carry molecules which enhance tissue- and organ-specific regeneration or wound healing. This suggests that they can also be utilized for drug delivery [220,221,222,223]. As the protein profile of EVs changes depending on physiological conditions and environmental changes, EVs with a higher capacity for regenerative support may be produced by isolation or harvest under these provoking conditions. It is also possible to ‘load’ EVs with miRNA, phytochemicals, or some small drug compounds for the treatment of wounds and other disease processes. Methods of loading include incubation, transfection, in situ assembly, and synthesis [224,225,226]. An important question, then, is ‘what to load’. What would be the most effective molecules or particles to load to enhance regeneration?

In the previous section, we described some examples of the combinatorial use of multiple phytochemicals. It is plausible to use EVs and phytochemicals together as well by loading phytochemicals into mammalian EVs or PDEVs. Sun et al. (2010) loaded the phytochemical compound curcumin into EVs isolated from mammalian T lymphoblast cells EL4 [227] using a passive loading procedure, incubating curcumin with exosomes isolated from EL4 cells in PBS buffer at 22 °C for 5 min. Curcumin-containing exosomes were found to have higher chemical stability, bioavailability, and anti-inflammatory effects compared to exosome alone and curcumin alone in the in vitro assays using RAW 264.7 cells exposed to lipopolysaccharide (LPS) [227].

Another possibility is to load specific miRNAs that match the target. Pomatto et al. (2019) [228] used a transfection method to load miRNAs into plasma-derived EVs. The following miRNAs were selected for specific anti-tumor effects: cel-39-3p, miR-31-5p, and miR-451a [216]. EVs loaded with the miRNAs had significantly higher suppressing effects on the targeted genes related to anti-apoptotic pathways and increased apoptosis of HepG2 hepatocellular carcinoma cells [228]. This is a successful example of harnessing the target-specific nature of microRNAs and the delivery capacity of EVs. 

## 5. Limitations

We have summarized the issues that require consideration and improvements. Here, in Table 5, we summarize the merits and demerits of phytochemicals. The strength of phytochemicals, as a whole, is the extremely large number of phytochemicals with diverse bioactive properties. Large amounts of material are available if necessary. Many phytochemicals share receptors, and many activate multiple simultaneous pathways. Receptors may be expressed in various locations throughout the body, and, as a result, effects may be seen in multiple areas or systemically, which may be advantageous or problematic and undesired. Arguably, the largest problem with phytochemicals is their hydrophobic nature, which limits bioavailability and necessitates larger amounts to obtain the desired results. Advances in bioengineering and chemical engineering techniques have largely overcome these issues through the combinatorial use of different phytochemicals with similar bioactive properties, formulating inclusion complexes using, for example, cyclodextrin [229,230] or nano-emulsion technology [231,232,233]. The long-term stability of phytochemicals also poses a problem. BCP, for example, becomes air-oxidized to 50% in 5 weeks, and the oxidated product, caryophyllene oxide, is an allergen at moderate levels [234]. Linalool and limonene are known to often cause contact allergies [235]. Formulation not only enhances bioavailability, but it may also suppress oxidation by chemically trapping the phytochemical compounds, suppressing exposure to oxygen. Enhanced bioavailability can reduce the amount necessary to expect beneficial effects, potentially reducing adverse events. This may be a benefit of loading them into EVs as well, if they are molecularly small enough to do so.

In the case of EVs, the largest limitations may be the small cargo volumes and the difficulty in obtaining large volumes for therapeutic effect. The targets of microRNAs are more specialized than phytochemicals, suggesting they are more goal oriented. This can be beneficial if there are specific effects to be addressed and detrimental if the targets are broad in range. Another limitation may be the challenges observed in the recovery after storage of EV [236]. A recent study indicated that storing EV at −80 °C with PBS buffer supplemented with human albumin and trehalose improved the recovery [237].

**Table 5:** Summary of phytochemicals and EVs.

	Pros	Cons
Phytochemicals	Extremely large number of phytochemicals with bioactive properties. Depending on the phytochemicals, there are differences or overlap in the receptors they activate. Many phytochemicals activate multiple receptors and, thus, multiple signaling pathways. The receptors are often expressed in many tissues and organs.	Many are hydrophobic. Some are chemically unstable and become oxidized; oxidized products may have fewer effects and/or have side effects such as causing allergic reactions. Some can be allergens, causing contact allergies, depending on the person.
EVs	Specialized target: goal oriented	Only small volumes can be isolated. The method to isolate is not clearly established and the primary method using ultracentrifuge takes time. Possible involvement in allergic reaction [238] (Hovhannisyan et al. 2021)

These comparisons make it challenging to choose and combine the various chemicals and EVs so that they compensate for each other’s positive and negative attributes. A combination of mammalian EVs such as MSC-EV or SC-EV with phytochemicals such as BCP may produce synergistic effects by suppressing inflammation and enhancing cell proliferation and cell migration, thus facilitating regeneration in those cells and tissue types that express CB2 or other prominent BCP receptors. Loading PDEV with phytochemicals such as curcumin or EGCG and theaflavin may produce functions of extended duration through the prevention of oxidation. These possibilities need to be tested. Compared to the seemingly specific goal-oriented characteristics of the microRNAs present in or added to mammalian EVs, the effects of phytochemicals are expected to be more widespread due to the broad range of target receptors and the broad range of locations where the receptors are expressed. Together, they could be an ideal agent to assist in the recovery of injured tissues or organs.

## 6. Conclusions and Future Perspectives

As we have described above, phytochemicals with bioactive properties frequently facilitate wound healing and suppress inflammation. EVs also have similar capabilities. The cargo ship-like structure of EVs enables transport and transfer of nanoparticles like microRNAs and hydrophobic molecules like phytochemicals. Bioengineered EVs hold much promise as a delivery system for future therapeutics.

## Figures and Tables

**Table 1:** Examples of phytochemicals with anti-inflammatory effects.

Name CID CAS	Receptors/Channels and Signaling Pathways	Plants	Effects Other than Anti-Inflammation	Reference
Carnosic acid CID: 65126 CAS: 3650-09-7	PPAR γ [73]	Salvia, rosemary	Neuroprotective (review [74]); Alzheimer’s disease, Parkinson’s disease, long-COVID (review [75])	[73,74,75]
Carvacrol CID: 10364 CAS: 499-75-2	TRPA1, TRPV3 [76], PPARα and PPARγ [77], GABA_A_ (in mice [78]) Antagonist to nicotinic acetylcholine receptors (nAChRs) [79], antagonist to TRPM7 [80,81]	Oregano, thyme		[76,77,78,79,80,81]
β-caryophyllene CID: 5281515 CAS: 87-44-5	CB2 [36]; PPARα directly [46] and PPARγ indirectly [47,48,49]	Copaiba, black pepper, rosemary and others (review [82])	Anti-carcinogenesis [83], analgesic [40,84], facilitates wound healing, cell proliferation/migration (in vivo in mice and in vitro [17])	[17,36,40,82,83,84]
Citral (geranial) CID: 638011 CAS: 5392-40-5	GABA_A_, 5-HT_1A_ [85]; TRPV1, TRPM8 [86]	Lemon grass (*Cymbopogon citratus*), lemon	Anti-nociceptive (in mouse and rat model) [86]; anxiolytic (in mouse model) [85]	[85,86]
Curcumin CID: 969516 CAS: 458-37-7	Potential ligand of Aryl hydrocarbon (AhR) [70], GPR55 [87], PPARγ [69,88], TRPA1 [71,89], and others	Turmeric (*Curcuma longa*)	Anti-carcinogenesis, anti-viral, antiarthritic, anti-amyloid, antioxidant [72]	Review, in vitro, and in vivo animal studies [69,70,71,72,87,88,89]
Epigallocatechin-3-gallate (EGCG) CID: 65064 CAS: 989-51-5	67LR (67-kDa laminin receptor) [90,91]	Tea plant (*Camellia sinensis*)	Antiviral [92,93], antibacterial [94], anti-oxidant, anti-cancer [95,96]	In vitro studies and reviews [90,91,92,93,94,95,96]
Eugenol CID: 3314 CAS: 97-53-0	TRPV1 [97]; PPARγ [98]	Clove	Antidiabetic, antioxidant, anti-cancer [98]	In vitro study and a study using *C. elegans* [97,98]
Gingerol = group of phytochemicals such as 6-gingerol (CID 442793; CAS 23513-14-6), 10-gingerol (CID 168115, CAS 23513-15-7), and others	TRPV1 [99]	Ginger	Anti-oxidant, antibacterial, anti-cancer [99]; immunomodulatory, neuroprotective, respiratory protective, antiobesity, antidiabetic, antinausea [100]; antiallergic [101]	Reviews [99,100,101]
D-Limonene CID: 440917 CAS: 5989-27-5	A2A adenosine receptor [102,103]; TRPA1 [104]	Bitter orange	Anti-carcinogenesis; anxiolytic [105]; topical application cause pain (algesic) through TRPA1	In vivo and in vitro studies [102,103,104,105]
Linalool CID: 6549 CAS: 78-70-6	GABA_A_ [106]; hypothalamic orexin neurons involved [107,108]	Mint, lavender	Anxiolytic effects [106]; analgesic [108]; antinociceptive	In vivo studies using mice [106,107,108]
Perillyl alcohol CID: 10819 CAS: 536-59-4	Ras/MAPK pathway inhibitor [109]	Lavender, citrus fruits, peppermint, spearmint, cherries	Anti-carcinogenesis [110,111], anti-nociceptive [112]	In vivo, in vitro, and review [109,110,111,112]
Resveratrol CID: 445154 CAS: 501-36-0	Estrogen receptor α [113,114,115], TAS2R50 (bitter taste-sensing receptor) [116], PPARα and PPARγ antagonist [117]; but another study reports PPARγ agonist [118]	Grapes, peanuts, blueberries, cranberries	Amti-carcinogenesis [115], antiviral, antioxidant	In vitro studies and reviews [113,114,115,116,117,118]
Theaflavin CID: 135403798 CAS: 4670-05-7	Bitter taste receptor hTAS2R39 hTAS2R14 [119]	Tea plant (*Camellia sinensis*)	Anti-viral [92], antibacterial, anti-diabetic, anti-cancer, [120,121]	In vitro and reviews [92,119,120,121]

**Table 3:** Combinatorial use of multiple phytochemicals and single use of them (studies are not included if more than two phytochemicals were mixed or phytochemicals were mixed with chemotherapy drugs). All are in vitro studies.

Phytochemicals	Effects Found	References
Curcumin	Administration of curcumin suppressed expression of proinflammatory cytokines TNFα, IL-1β, and NF-kb and the expression of glial fibrillary acidic protein, suggesting reduced glial scar formation, in a rat model of spinal cord injury [167]. Transgenic mice with autoimmune desease (NZBWF1) administered with curcumin showed less renal injury [168]	[167,168]
Piperine	Piperine possesses anti-inflammatory effects, neuroprotective effects, anti-microbial effects, and anti-cancer effects (review). It also has bioavailability-enhancing effects (review). Piperine also suppressed cytochrome P450 in a mouse model [169] and had hepatoprotective effects on thioacetamide-induced liver fibrosis in mice [170]. Piperine administration suppressed the experimentally induced pancreatitis by administration of cerulein in mice [171]	[169,170,171,172]
Resveratrol	Anti-inflammatory effects and neuroprotective effects in rats with cuprizone-induced demyelination [173]	[173]
EGCG	Anti-viral effects on SARS-CoV-2 [58,92], anti-bacterial effects [94], anti-cancer effects [95]	[58,92,94,95]
Quercetin	Anti-inflammatory effects, antioxidant effects, suppression of nociceptive and pathological pain (review [174]). Quercetin suppressed COX-2 expression in RAW264.7 cells inflamed with lipopolysaccharides (LPS) [175]	[174,175]
Curcumin + piperine	Co-administration improved the lipid profile of metabolic syndrome [163]; suppressed cancer cell proliferation through inhibitory effects on Wnt signaling mTORC1 signaling [164]	[163,164,165]
Curcumin + EGCG	Suppresses cancer cell proliferation	[164,166,176,177]
Curcumin + resveratrol	Anti-cancer; suppresses cancer cell survival	[164]
Curcumin + quercetin	Suppresses cancer cell survival	[164]
